# Lentivirus-Carried microRNA-195 Rescues Memory Deficits of Alzheimer’s Disease Transgenic Mouse by Attenuating the Generation of Amyloid Plaques

**DOI:** 10.3389/fphar.2021.633805

**Published:** 2021-04-26

**Authors:** Dan Su, Yani Chai, Junkai Yang, Xuqiao Wang, Ying Liu, Jing Ma, Xin Tang, Chandan Mishra, Shah Ram Chandra, Weidong Yue, Jing Ai

**Affiliations:** ^1^ Department of Pharmacology (The State-Province Key Laboratories of Biomedicine-Pharmaceutics of China), College of Pharmacy of Harbin Medical University, Harbin, China; ^2^ Department of the 2nd Affiliated Hospital of Harbin Medical University, Harbin, China

**Keywords:** miR-195, APP/PS1 transgenic mice, Aβ plaques, cognition, multiple molecular targets

## Abstract

Although lots of new drugs are developed to treat Alzheimer’s disease (AD), many clinical trials of monotherapy have failed to affect disease progression or symptoms compared with placebo. Recently, scientists believe that combination treatment is more promising than monotherapy. Previous studies found that microRNA-195 (*miR-195*) was down-regulated in the hippocampi and cortices of chronic brain hypoperfusion (CBH) rats and ApoE4^(+/+)^ mice, and up-regulation of *miR-195* can improve the declined cognitive function of ApoE4^(+/+)^ mice and CBH rats by targeting multi-genes that are related to AD pathology, including amyloid precursor protein (APP) and β-site APP cleaving enzyme 1 (BACE1) genes. However, whether the gain-of-function of *miR-195* could improve the impaired learning and memory ability of APP/PS1 transgenic mouse has not been reported. In this study, we stereotaxically injected lentiviral-carried *miR-195* into the bilateral hippocampus of 4-month-old (4M) APP/PS1 mice. Morris water maze (MWM) was performed to detect the effect of miR-195 on the cognitive function of APP/PS1 mice after 1M, 2M, and 3M treatment. Western blot was used to detect the expression of APP, BACE1, and AT8. Aβ plagues were quantitatively assessed by immunofluorescence technique. We found that the declined cognitive phenotype of APP/PS1 mice occurred at the age of 6M, not at the age of 5M. And treatment of Lv-pre-*miR-195* to APP/PS1 mice for 1M did not achieve any changes. Although Lv-pre-*miR-195* treatment for 2M improved the declined learning ability of APP/PS1 mice, it did not affect the memory functions. However, Lv-pre-*miR-195* treatment in APP/PS1 mice for 3M can effectively improve both the learning and memory ability of APP/PS1 mice at the age of 7M. Further studies demonstrated that gain-of-function of *miR-195* by Lv-pre-*miR-195* injection could inhibit the increased APP and AT8 expression of APP/PS1 mice but did not affect BACE1 level that was not changed in both hippocampus and cortex. By counting the number of Aβ plaques of different sizes, we found that Lv-pre-*miR-195* treatment mainly reduced the number of Aβ plaques of less than 20 μm, but did not affect the number of Aβ plaques of greater than 50 μm. Taken together, the gain-of -function of *miR-195* in the hippocampus can improve the cognition of APP/PS1 mice, probably by blocking the formation of Aβ plagues rather than clearing those that have already formed Aβ plagues.

## Introduction

Alzheimer’s disease (AD) is typically characterized by senile plaques because of deposition of Aβ peptide, neurofibrillary tangles by hyperphosphorylation of Tau protein, and brain atrophy due to neuron loss (Hardy, 2009; Henry et al., 2013; Busche and Hyman, 2020). Based on this consensus, many precise targeting specific protein molecules have entered clinical trials, these drugs including BACE1 inhibitor, γ-secretase inhibitor, Aβ aggravation blocker, tau stabilizers and aggregation inhibitor, and anti-tau and anti-Aβ immunotherapy, et al. (Dodel et al., 2003; Godyn et al., 2016; Panza et al., 2019). So far, however, compared with placebo in clinical trials, these monotherapies have failed to affect disease progression or symptoms (Dodel et al., 2003; Godyn et al., 2016; Panza et al., 2019). Recently, the scientists believe that combination therapy might be better than monotherapy (Bachurin et al., 2017; Sahoo et al., 2018; Cummings et al., 2019; Schaduangrat et al., 2019). However, the complex pathophysiology of AD makes the design of combination therapy more difficult. Finding a molecule that targeting multiple molecular targets simultaneously would be more effective than combining multiple drugs.

MicroRNAs have been well known for targeting multiple molecular targets simultaneously and participating in multiple physiological and pathological processes. They are considered as drug targets for the RNA-based therapeutic strategies (Lu and Thum, 2019; Revia et al., 2019; Brennan and Henshall, 2020). Our previous study found that microRNA-195 (*miR-195*) was down-regulated in the hippocampus and cortex of rats suffering from chronic brain hypoperfusion (CBH) (Ai et al., 2013), which was recently verified in the brain tissue of patients with the clinical diagnosis of mild cognitive impairment (MCI) (Cao et al., 2020). Thereafter, a series of studies demonstrated that down-regulation of *miR-195* targets multiple genes and participates in a variety of pathological processes under CBH status by up-regulating APP and BACE1 (Ai et al., 2013); promoting hyperphosphorylation of Tau by increasing p35 level and activating calpain to accelerate the conversion of CDK5/p35 to CDK5/p25; inactivating PP2A by elevating PME-1expression to inhibit methylation level of PP2A_C_ at Leu309 residue (Sun et al., 2015; Liu et al., 2016); facilitating dendritic remodeling and neuron death through promoting N-APP expression and post-transcriptionally up-regulating DR6 which could induce the activation of caspases 3 and 6 (Chen et al., 2017). Furthermore, elevating *miR-195* ameliorated cognitive deficits, amyloid plaque burden, and tau hyper-phosphorylation in ApoE4 (+/+) mice and rescued AD-related lysosomal defects in inducible pluripotent stem cells (iPSCs)-derived brain cells of ApoE4 (+/+) AD patients, while inhibiting *miR-195* exacerbated these phenotypes by targeting synaptojanin 1 (synj1) (Cao et al., 2020). These results suggest that *miR-195* might be a potential multi-target drug to prevent or treat AD at the early stage. However, whether *miR-195* has enough effect to rescue the cognitive decline of an animal model of familial AD is unclear.

APPswe/PS1dE9 (APP/PS1) transgenic mouse is a kind of double transgenic mice expressing a chimeric mouse/human amyloid precursor protein (Mo/HuAPP695swe) and a mutant human presenilin 1 (PS1-dE9), both directly transfected to the CNS neurons to allow the mice to secrete a human A-beta peptide (Aβ). APP/PS1 mouse was proved to be associated with early-onset AD and is a good animal model to evaluate efficiency of candidate drugs. In the present study, we first reported that lentiviral vector-mediated *miR-195* effectively rescued the cognitive decline of APP/PS1 mice by preventing the generation rather than eliminating the deposition of Aβ plaques.

## Materials and Methods

### Animals

Male APPswePS1dE9 mice (1∼4 months old purchased from Beijing HFK Bioscience CO.LTD.) were housed in the animal feeding room. Humidity was maintained at 55 ± 5% and the temperature was kept at 23 ± 1°C. The room is maintained under a 12 h light/dark cycle (lights on at 7:00 A.M). Animals for brain injection surgery were anesthetized with 10% sodium amobarbital (100 mg kg^−1^) by intraperitoneal injection. Samples for immunofluorescence staining, quantitative real-time (qRT)-PCR and western blot assay were obtained from the hippocampus and/or cortex of mice after they were anesthetized with sodium pentobarbital (100 mg kg^−1^) followed by confirmation of death by exsanguination. All animal procedures were approved by the Institutional Animal Care and Use Committee at Harbin Medical University (No. IRB3007719) and the Institute of Laboratory Animal Science of China (A5655-01). All procedures were conformed to the Directive 2010/63/EU of the European Parliament.

### Stereotaxic Injection of the Lentiviral Vectors

After anesthesia, APP/PS1 mice at the age of 4 months were placed onto a stereotaxic frame (RWB Life Science Co. Ltd., China). Injection coordinates relative to the bregma were as follows: AP (anteroposterior), −3.8 mm; ML (mediolateral), ±1.7 mm; DV (dorsoventral), −1.8 mm below the surface of dura using coordinates derived from the atlas of Paxinos and Watson. One and a half microliters Lv-pre-*miR-195* (2.85 × 10^8^ TU ml^−1^) or Lv-NC per hemisphere was injected into the CA1 of bilateral hippocampus by a stereotaxic frame with a 5 μL Hamilton syringe with a 33 gauge tip needle (Hamilton) at the rate of 0.3 μL/min^−1^ (Ma et al., 2015). The needle was then maintained in the place for 3 min and withdrawn very slowly to avoid liquid reflux before moving to the other site. After the injection, animals were used for different experiments after raising additional 1 month, 2 months, and 3 months, respectively.

### Construction of Lentivirus Vectors

Based on the lentivirus system of human immunodeficiency disease, the vector skeleton of the miRNA precursor expression clone was constructed. Lentivirus vector was composed of a lentivirus genome sequence and bacterial plasmid sequence. Highly purified plasmids, EndoFectin-Lenti™ and TiterBoost™ reagents were used in the process of the lentiviral particles. Cloning of miExpress™ precursor miRNAs expression using eGFP as reporter gene clone the stem-loop precursor of *miR-195* into the virus. The lentiviral transfer vector was co-transfected into 293T cells (Cat #: CLv-PK-01) with Lenti-Pac™ HIV packaging mix (Cat#: HPK-LvTR-20). The plasmid sequence of bacteria contained the Ampicillin resistance gene and a high copy replicon PUCori. The sequence of lentivirus genome elements started with 5′LTR and ended with 3′LTR. The pre-*miR-195* gene precursor sequence was designed as: aca​ccc​aac​tct​cct​ggc​tct​agc​agc​aca​gaa​ata​ttg​gca​tgg​gga​agt​gag​tct​gcc​aat​att​ggc​tgt​gct​gct​cca​ggc​agg​gtg​gtg​a. We cloned the oligonucleotides into the pEZX-MR03 vector. The vector was identified after analyzing the plasmid sequence (Invitrogen). The titers of Lv-pre-*miR-195* used for experiments were 2.85 × 10^8^ TU/ml. The virus was stored at −80°C until use and was centrifuged and kept on ice before injection.

### Morris Water Maze

The pool of the Morris water maze of 1.2 m diameter had a submerged platform of 9 cm diameter which was located in the first quadrant. Two days before the experiment, water was poured into the pool to balance the water temperature, and the water level was about 2 cm above the platform. All mice were tested for pupillary light reflex before training, and the mice with impaired pupillary light reflex were excluded from the experiment, to avoid the effect of animal vision on the test. The mice were trained in the Morris water maze for 5 days and accessed to the water in turn from three quadrants except for the quadrant where the platform was located in every day. If they arrived at the platform within 120 s, the experiment was stopped. If they were not on stage at the specified time, they were guided to stand on the platform. On the day 6, the platform was taken out in advance. The mice were put into the pool from the opposite quadrant of the platform, and the number of times of passing through the platform site was recorded (Ma et al., 2015). The online Dig Behav-Morris water maze video analysis system (Mobile Datum Software Technology) was used to monitor the latency, swimming path length (cm), swimming speed (cm s^-1^), the number of times to cross the platform, and the percentage of swimming distance in the target quadrant to the total distance of the swimming pool. The heat map was a program made by Matlab based on the spatial distribution probability of the path and judging the time of the mouse moving in different positions by the color value. Behavioral testing was conducted at the 6 months or 7 months of age that were 2 or 3 M after surgery, respectively. All behavioral testing and data analysis were conducted under double-blind conditions.

### Immunofluorescence Staining

The brains were fixed with 4% paraformaldehyde overnight at 4°C and dehydrated with 30% sucrose until they sunk to the bottom of the fluid. Brain slices were cut into slices at 30 μm thickness. The slice we selected for the quantitative analysis was the site of bregma −3.16 to −2.92 mm and interaural 0.64–0.72 mm according to mouse brain atlas. The slices were incubated with penetrating solution at room temperature for 1.5 h then blocked with Triton X-100 and 10% goat serum (Mao et al., 2020). After slices were incubated with the primary antibodies anti-β-Amyloid (D3D2N) mouse mAb (1:200, #15126, CST, United States), AT8 (1:50∼1:1000, #MN1020B, Invitrogen™, Carlsbad, CA, United States) overnight at 4°C, followed by secondary antibodies Alexa Fluor 488 or Alexa Fluor 594 (1:200, Molecular Probes, Eugene, OR, United States) for 1 h as well as DAPI (1:200,#C1002,Beyotime, China) staining for 15 min at the next day. Finally, confocal images were captured by high resolution panoramic imaging and analysis system (Leica Biosystems, aperio versa 8, Germany) under the control of Aperio Image Scope software (Leica Biosystems, Germany).

The fluorescent brain slice images were analyzed and measured by Image pro Plus software. After setting the image scale, the color pickup tool was used to select the mouse plaque area of interest. Based on the counting function, the plaque area can be segmented and counted automatically. The area of the mouse brain can be measured based on the manual polygon circle.

### Western Blot Analysis

Tissue samples were extracted from hippocampus and temporal lobe of cortex. The protein concentrations of all extracts were measured by Bio-Rad protein analysis (Bio-Rad) combined with bovine serum albumin standard. Target protein antibody included: APP (1:1000, ab92305, Abcam, MA, United States), BACE1 Rabbit Recombinant mAb (1:1000, A5095, Bimake, United States), AT8 (1:1000, #MN1020B, Invitrogen™, Carlsbad, CA, United States), and β-actin (1:1000, G8795, Sigma, Saint Louis, MO, United States) diluted with PBS. For APP and AT8 proteins, the amount of protein sample was 20 μg and that was 40 μg for BACE1. After being fractionated with SDS-PAGE gel, they were transferred to the PVDF membrane. Membranes with antibodies were put into the shaker at 4°C for overnight incubation. The next day, the antibody was washed off with PBST solution, and the second antibody was added. After 40 min, the second antibody was washed off with PBST solution. Western blot bands were scanned by Odyssey Infrared Imaging System (LI-COR Bioscience, Lincoln, NE) and analyzed by Odyssey v. 1.2 software which normalized to the internal control, β-actin (Ai et al., 2013).

### Quantitative Real-Time PCR

Total RNA was purified with the Trizol Reagent (Invitrogen, Carlsbad, CA, United States), according to the manufacturer’s instructions. SYBR Green PCR Master Mix Kit (Applied Biosystems, Foster City, CA, United States) was used for measuring the level of *miR-195* with U6 as an internal control. For measuring *miR-195*, the primersforward: 5′-ACACTCCAGCTGGGTAGCAGCACAGAAATATTG-3′,reverse: 5′CTC​AAC​TGG​TGT​CGT​GGA-3′ were used. The qRT-PCR was carried out on a 7500 fast real-time PCR system (applied biological system), and the protocol was as follows: 1) 95°C, 10 min; 2) 95°C, 15 s; 3) 60°C, and 1 min (repeat 2) and 3) for 40 cycles). The results were normalized with the internal control using the δ-δCT method (Sun et al., 2018).

### Data Analysis

Data were described as mean ± SD. Day-by-day between group’s comparisons was performed using factorial ANOVA (Split-plot design). Post hoc analyses of significant main effects were further examined using Fisher’s TURKEY tests. The two-tailed Student’s t-test was applied for comparisons between the two groups. *p* < 0.05 was considered statistically significant. For non-normally distributed data, chi-squared test was used to compare the difference between two groups. SPSS 9.1 software (Serial number: 989155. Institute Inc. China) was used for all statistical analyses. The graphs were generated by GraphPad Prism 5.0 software (La Jolla, CA, United States).

## Results

### Evaluation of Amyloid Plaques (Aβ) Deposition in the Hippocampus and Cortex of APP/PS1 Mice

**FIGURE 1:**
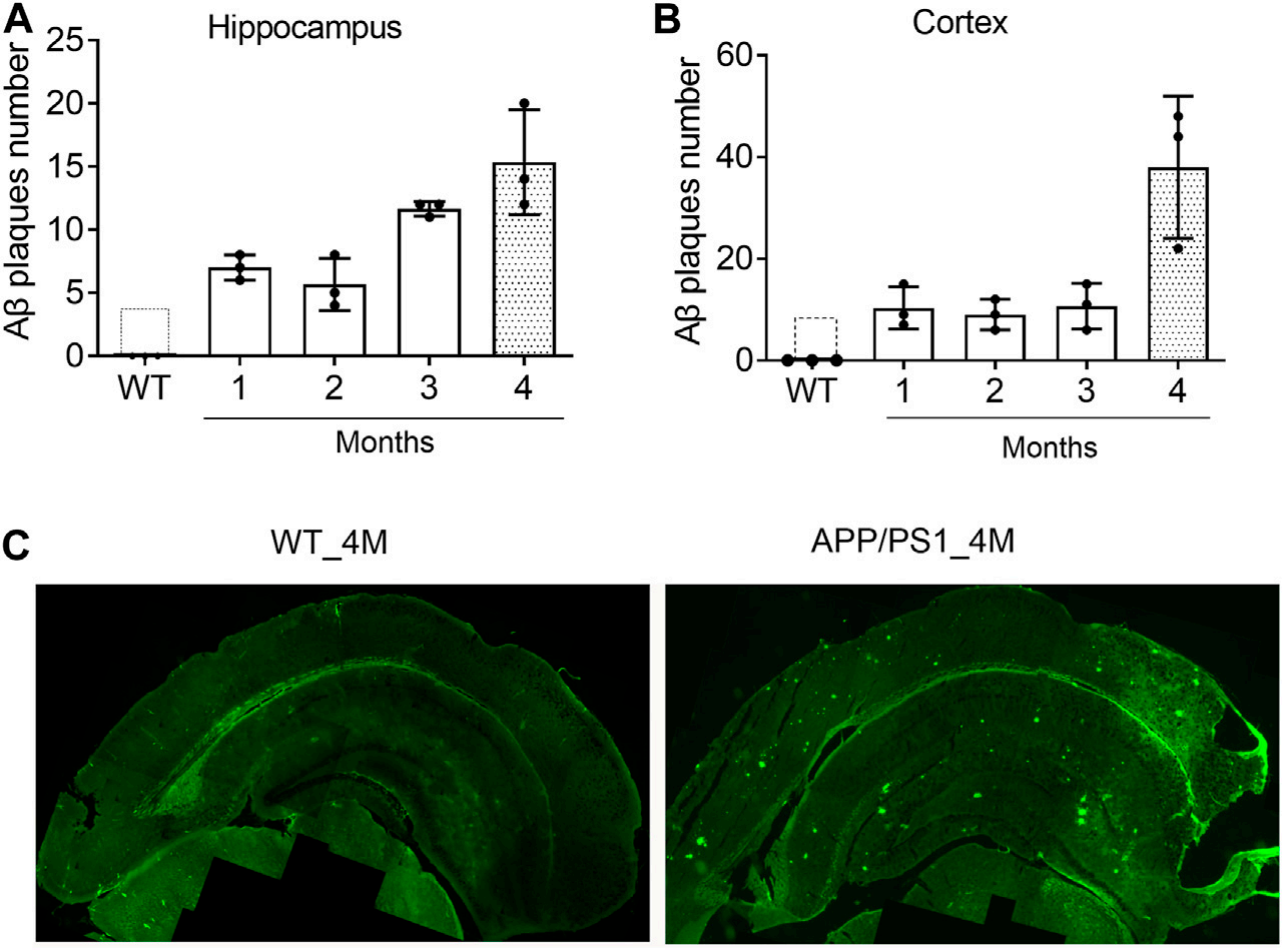
The deposition of Aβ plaques in the brain of APP/PS1 mice are rapidly increased from the age of 4M **(A and B)** The changes of Aβ plaques in the hippocampus and cortex of APP/PS1 mice at the age of 1–4M old. WT: wild type mice at the age of 4-months **(C)** Immunofluorescence image shows Aβ plaques in the brain of 4M-old APP/PS1 and WT mice.

Since one of the functions of *miR-195* is to inhibit the generation of Aβ in the brain of rats with chronic brain hypoperfusion (CBH) (Ai et al., 2013), APP/PS1 mice was selected as an animal model to evaluate the pharmacological effect of *miR-195*. To identify the optimal timing for drug intervention, we assessed the deposition of Aβ plaques in the brains of APP/PS1 mice from 1-month (1M) to 4M using immunofluorescence staining. We found that although APP/PS1 mice had Aβ plaque deposits in the hippocampus at 1M and 2M, the amount was much lower than APP/PS1 mice at 3 and 4M (Figures 1A,C). In the cortex of APP/PS1 mice, the number of Aβ plaques at the age of 4M was much higher than the age of 1–3M (Figures 1B,C), which was similar to previously reported (Aso et al., 2012). These results suggest that the Aβ plaques deposition in the brain of APP/PS1 mice increases rapidly from the age of 4M. Therefore, 4M of APP/PS1 mice were selected as the drug intervention time to evaluate the effect of *miR-195* on cognitive function.

### Lv-pre-*miR-195* Improves the Spatial Learning and Memory Ability of APP/PS1 Mice

**FIGURE 2:**
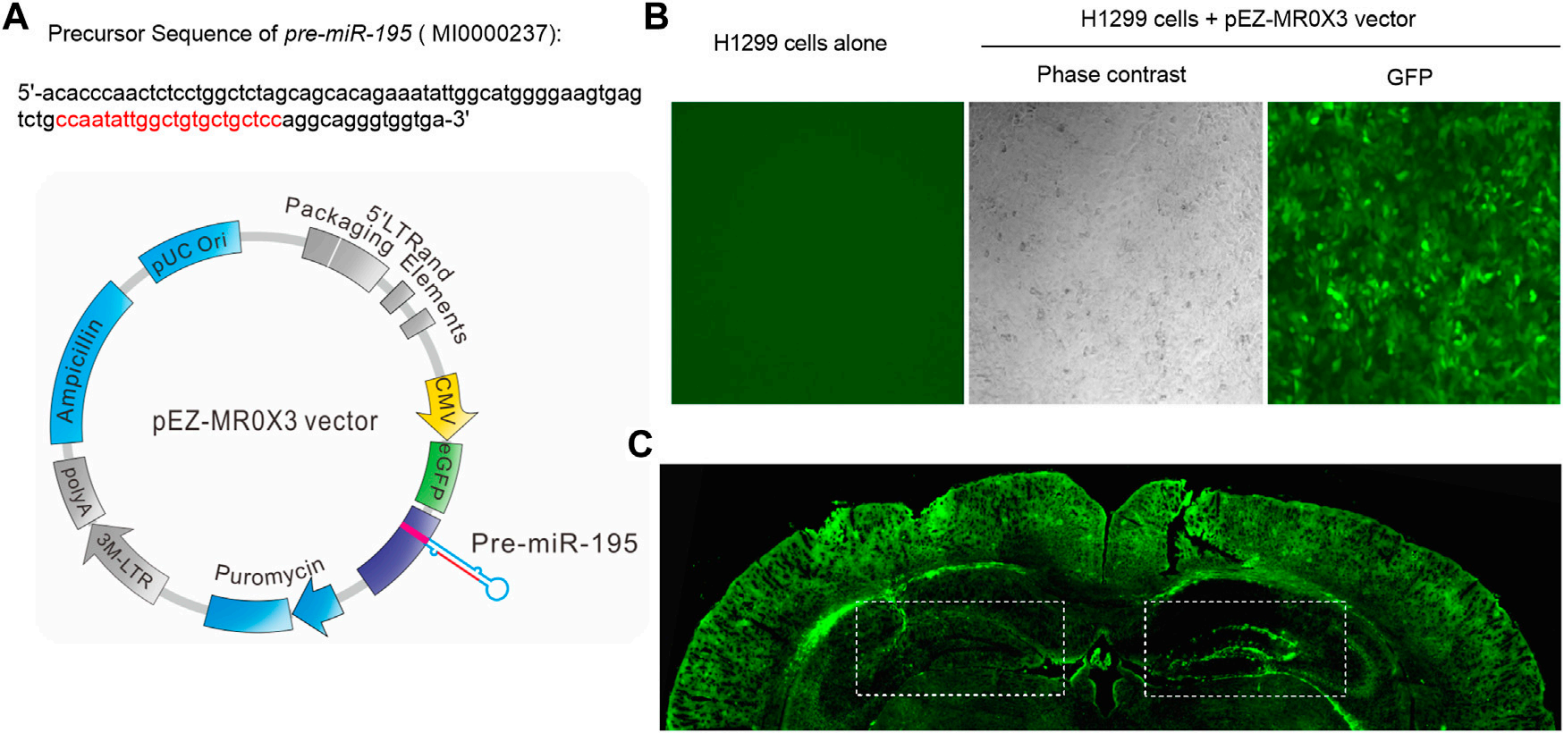
Plasmid construction and transfection efficiency evaluation **(A)** The structure of lentiviral vector containing mmu-pre-*miR-195* (Lv-pre*-miR-195*) and the precursor sequence of pre*-miR-195* **(B)** H1299 cells (in a 24-well plate) were transfected with 1 μL pEZ- MR0X3 vector for 3 days, and the expression of green fluorescent protein reporter gene was detected under fluorescence microscope **(C)** Green fluorescence was detected in the hippocampus and cortex of mice after cerebral injection of Lv-*pre-miR-195* for one month. The doses of lentivirus were 1 μL on the left side and 1.5 μL on the right side.

Previous study reported that knockdown *miR-195* in the hippocampus could induce the impairment of learning and memory ability (Ai et al., 2013; Cao et al., 2020). Reciprocally, up-regulation of *miR-195* by stereotaxic injection of lentiviral vector-mediated *miR-195* into the hippocampus could improve CBH induced cognitive decline in rats (Ai et al., 2013). Therefore, in the present study, we constructed lentiviral vectors containing mmu-pre-*miR-195* (Lv-pre-*miR-195*) using pEZ- MR0X3 (Figure 2A) and it can be successfully transfected into H1299 cells (Figure 2B). We then stereotaxically injected Lv-pre-*miR-195* into bilateral hippocampi with 1 μL on the left and 1.5 μL on the right (Figure 2C). After 1-month (1M) of injection, we found the transfection efficiency of the lentiviral vector using the dosage of 1.5 μL was better than that 1 μL injection dose (Figure 2C). Therefore, 1.5 μL lentiviral vector in each injection was performed in the follow-up experiments. Since the greatest advantage of lentiviral vectors is that they can achieve long-term stable expression, we decided to observe the action of Lv-pre-*miR-195* on cognition of APP/PS1 mice after Lv-pre-*miR-195* injection for 1M, 2M, and 3M.

**FIGURE 3:**
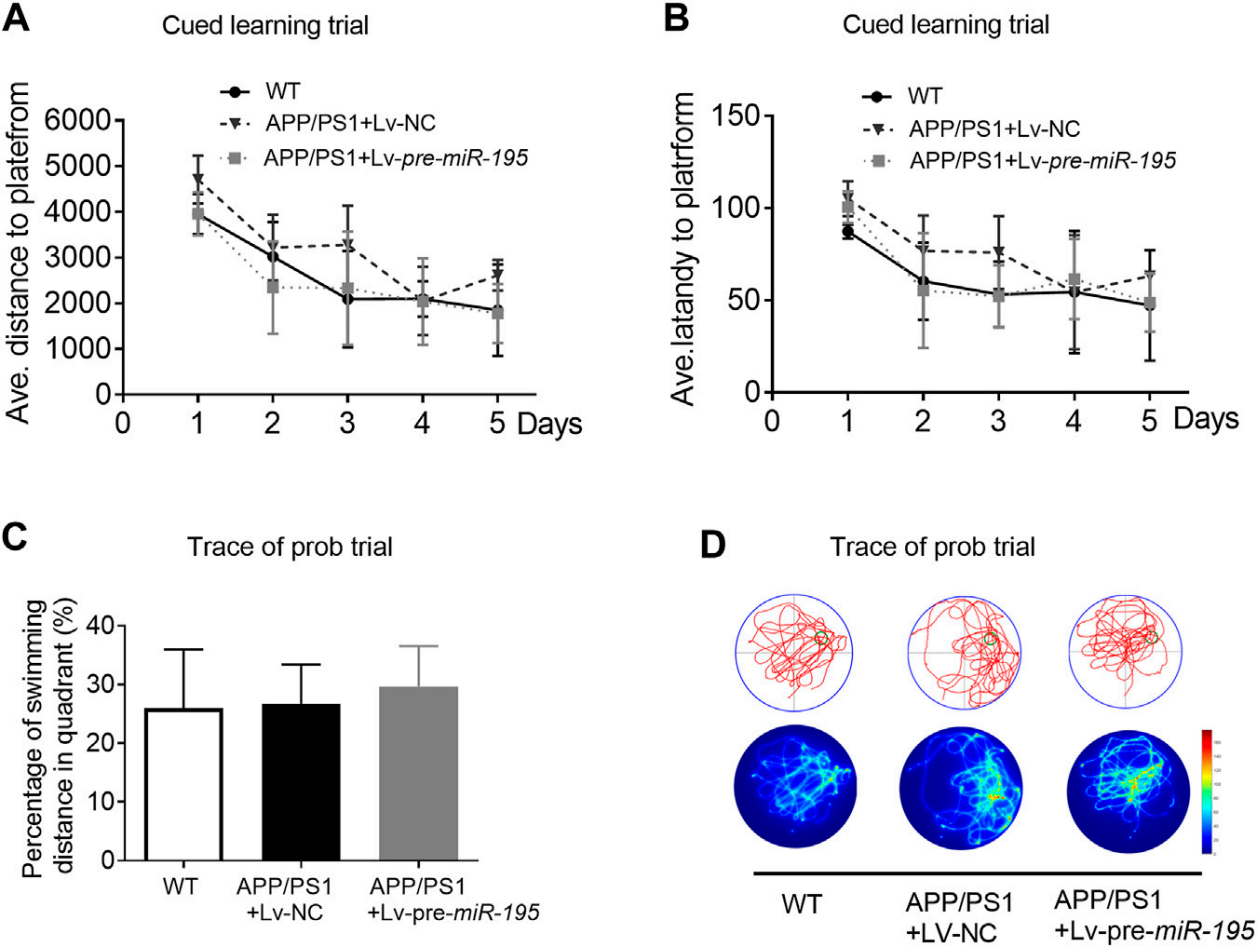
APP/PS1 mice at the age of 5M-old showed no significant spatial learning deficits **(A)** Average distance to platform after Lv-pre*-miR-195* injection for 1M. *n* = 6 for WT, Lv-NC and Lv-pre*-miR-195* group **(B)** Average escape latency to platform after treatment of Lv-*pre-miR-195* for 1M. *n* = 6 for WT, APP/PS1 and Lv-pre*-miR-195* group **(C)** Percentage of swimming distance in the target quadrant relative to the total distance of the pool during probe trial. *n* = 6 for WT, Lv-NC and Lv-pre*-miR-195* group **(D)** Representative path tracings and heat maps of the probe test on day 6 in the MWM test for each group.

We then used the Morris water maze (MWM) test to evaluate the ability of spatial learning and memory as previously reported (Vorhees and Williams, 2006; Ai et al., 2013). Similar to previous study (Psotta et al., 2015), the ability of spatial learning and memory of APP/PS1 mice at the age of 5M was not changed. Here we found no change in APP/PS1 mice after Lv-pre-*miR-195* treatment for 1 month (Figures 3A–D; post hoc analysis, A: *F*_(4,63)_ = 3.884, *p* = 0.057; B: *F*_(4,63)_ = 2.109, *p* = 0.164).

**FIGURE 4:**
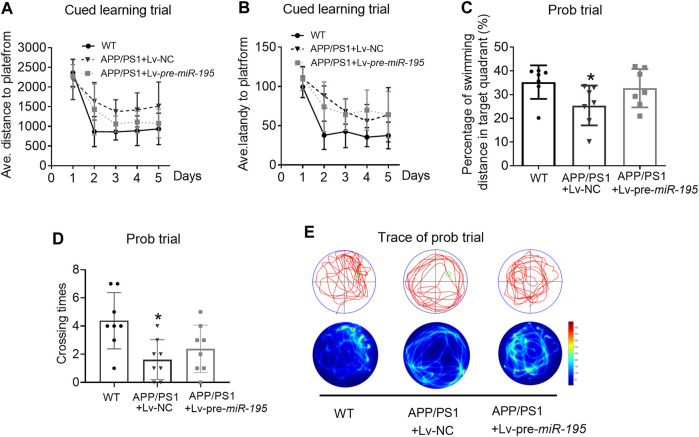
*MiR-195* attenuates the declined learning ability but not affects the impaired memory function in APP/PS1 mice after brain injection of Lv-pre-*miR-195* for 2M **(A)** Average distance to platform after brain injection of Lv-pre-*miR-195* for 2M. *n* = 8 for WT, Lv-NC and Lv-pre*-miR-195* group **(B)** Average escape latency to platform after treatment of Lv-pre*-miR-195* for 2M. *n* = 8 for WT, Lv-NC and Lv-pre*-miR-195* group **(C)** Percentage of swimming distance in the target quadrant relative to the total distance of the pool during probe trial. *p < 0.05 vs. WT, *n* = 8 **(D)** Number of times to cross the target platform during probe trial. *p < 0.05 vs. WT, *n* = 8 **(E)** Representative path tracings and heat maps of the probe test on day 6 in the MWM test for each group.

Thereafter, we evaluated spatial learning and memory in 6M old APP/PS1 mice vs. age-matched WT C57/B6 mice. As shown in Figure 4, in learning trial, we observed that the APP/PS1 mice took more distance (Figure 4A, For groups: *F*_(4,84)_ = 7.114, *p* < 0.005; WT *vs* Tg: *p =* 0.004) and more time (Figure 4B, For groups: *F*_(4,84)_ = 9.107, *p =* 0.002; WT *vs* Tg: *p =* 0.006) to find the hidden platform after they had been released into the tank in all of the three non-target quadrants day-by-day. The results indicated that APP/PS1 mice displayed the decreased ability to reach the target through the spatial location cues. Surprisingly, after 2M of Lv-pre-*miR-195* injection, both the average swimming distance (Figure 4A, Tg *vs* Tg + Lv-pre-*miR-195*: *p* = 0.138) and latency (Figure 4B, Tg *vs* Tg + Lv-pre-*miR-195*: *p* = 0.933) were significantly improved. In the probe trial, APP/PS1 mice showed a reduced percentage of swimming distance in the target quadrant relative to WT mice (Figure 4C, *p* = 0.035 *vs* WT) and decreased platform crossings than in the WT group (Figure 4D, *p* = 0.007 *vs*WT). However, Lv-pre-*miR-195* treatment failed to reverse the decreased percentage of swimming distance in the target quadrant (Figure 4C, *p* = 0.124 *vs* Lv-NC) and crossing times (Figure 4D, *p* = 0.350 *vs* Lv-NC). The result was further verified by the swimming trace recorded in the prob trial (Figure 4E). The data suggested that Lv-pre-*miR-195* treatment for 2M could improve the declined learning ability but not affected the impaired memory function of APP/PS1 mice at the age of 6M.

**FIGURE 5:**
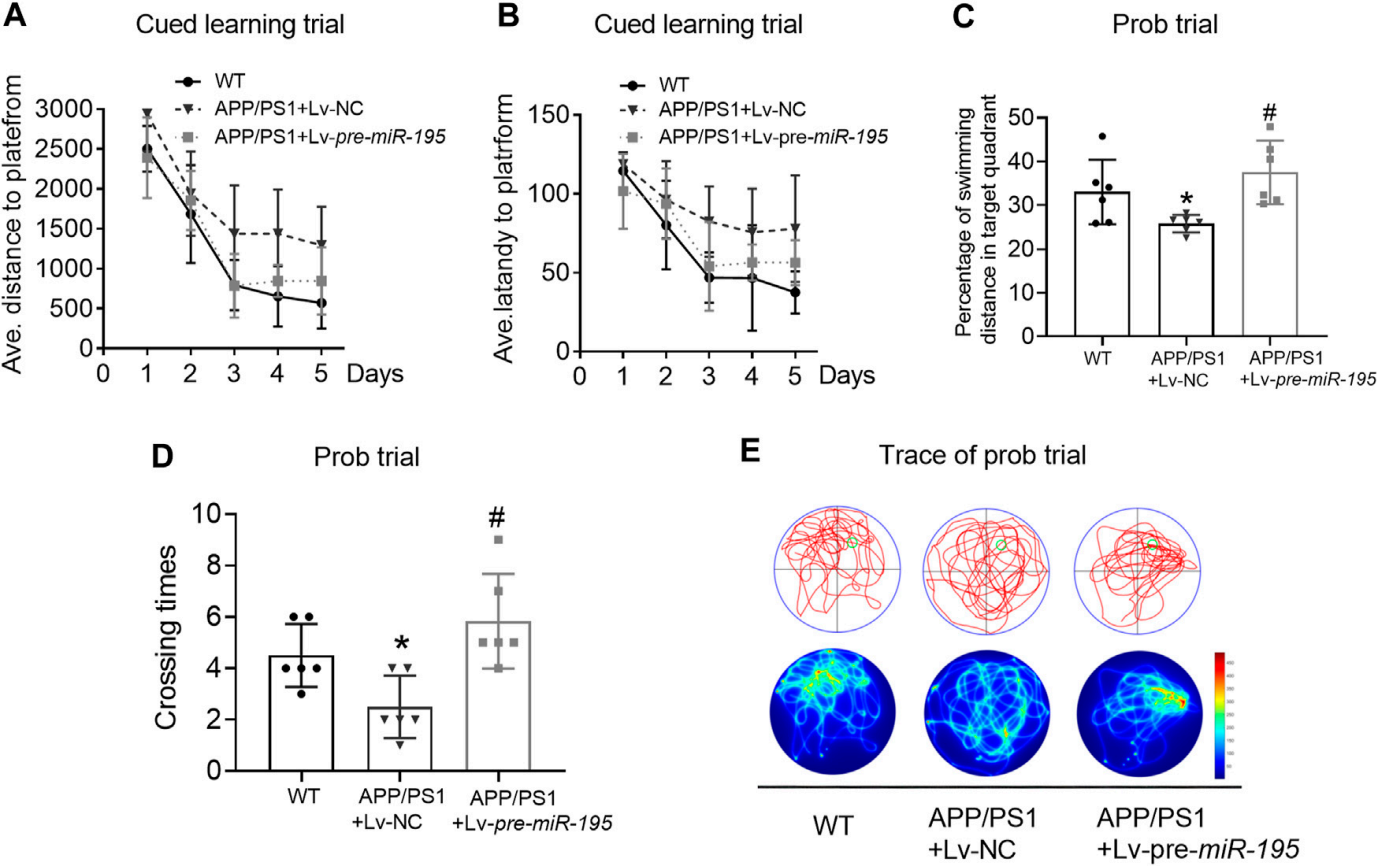
*MiR-195* attenuates the declined learning and memory in APP/PS1 mice after brain injection of Lv-pre-*miR-195* for 3M **(A)** Average distance to platform after Lv-*pre-miR-195* injection for 3M. *n* = 6 for WT, Lv-NC and Lv-pre*-miR-195* group **(B)** Average escape latency to platform after treatment of Lv-pre*-miR-195* for 3M. *n* = 6 for WT, Lv-NC and Lv-*pre-miR-195* group **(C)** Percentage of swimming distance in the target quadrant relative to the total distance of the pool during probe trial. *p < 0.05 vs. WT, *n* = 6. ^#^ *p* < 0.05 vs. Lv-NC, *n* = 6 **(D)** Number of times to cross the target platform during probe trial. *p < 0.05 vs. WT, *n* = 6. #*p* < 0.05 vs. Lv-NC, *n* = 6 **(E)** Representative path tracings and heat maps of the probe test on day 6 in the MWM test for each group.

We then extended the treatment time of Lv-pre-*miR-195* to 3M. Surprisingly, we found that, in the learning trial, Lv-pre-*miR-195* treatment effectively reversed the increased swimming distance of APP/PS1 mice (Figure 5A, For groups: *F*_(4,71)_ = 9.319, *p* = 0.002; WT *vs* Tg: *p =* 0.031; Tg*vs* Tg + Lv-pre-*miR-195*: *p =* 0.013), and the prolonged latency (Figure 5B, For groups: *F*_(4,71)_ = 4.419, *p* = 0.035; WT *vs* Tg: *p =* 0.031; Tg *vs* Tg + Lv-pre-*miR-195*: *p =* 0.162). Furthermore, in the probe trial, Lv-pre-*miR-19*5 injection also successfully improved the decreased percentage of swimming distance of APP/PS1 mice in the target quadrant (Figures 5C,E, *p* = 0.162 *vs* Lv-NC) and the elevated the crossing times (Figures 5D,E, *p* = 0.004 *vs* Lv-NC). The results suggest that Lv-pre-*miR-195* treatment with 3M can improve the impaired learning and memory ability of APP/PS1 mice at the age of 7M.

### Lv-pre-*miR-195* Treatment Prevents Aβ Deposition by Inhibiting APP Expression in APP/PS1 Mice

**FIGURE 6:**
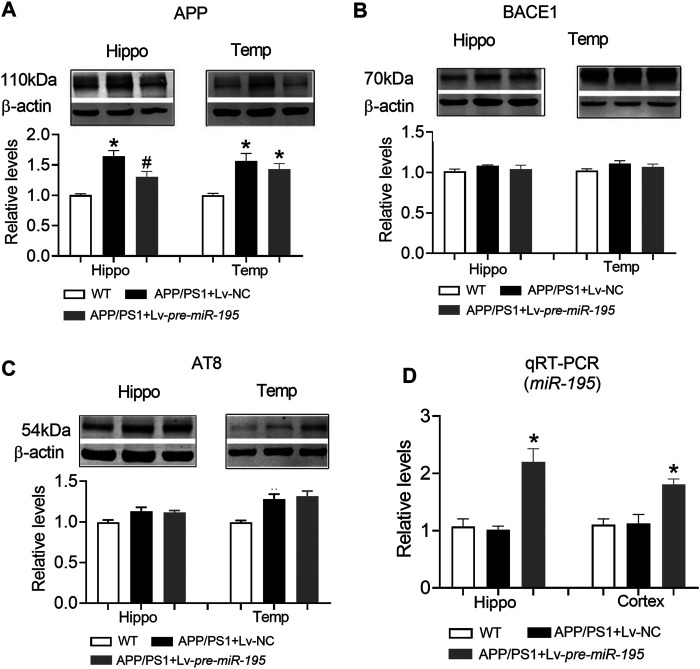
The effect of *miR-195* on the expression of APP, BACE-1, AT8 protein in the hippocampi of APP/PS1 mice after Lv-pre*-miR-195* injection for 2M **(A)** *MiR-195* down-regulated the expression of APP protein in hippocampus but did not affect it on temporal lobe cortex of APP/PS1 mice. *p < 0.05 vs. WT. #*p* < 0.05 vs. Lv-NC **(B)** There was no significant change in BACE-1 protein in hippocampus and temporal lobe of 6M-old APP/PS1 mice **(C)** *MiR-195* doesn’t reverse the high expression of AT8 in the brain of 6M-old APP/PS1 mice. *p < 0.05 vs. WT **(D)** *MiR-195* levels in the hippocampus and cortex of APP/PS1 mice after intracerebral injection of Lv-pre*-miR-195* 2M. *p < 0.05 vs. WT. *n* = 6.

Previous studies have shown that *miR-195* improves the impaired learning and memory ability of CBH rats by regulating the expression of APP and BACE1 at the post-transcriptional level (Ai et al., 2013). We hypothesized that Lv-pre-*miR-195* treatment improved cognitive performance in APP/PS1 mice through the same mechanism. As illustrated in Figure 6, we found that APP levels in both hippocampus and temporal cortex of APP/PS1 mice aged 6M were significantly increased (Figure 6A, hippocampus: *p* < 0.0001 *vs* WT; cortex: *p* = 0.001 *vs* WT), but BACE1 expression did not change (Figure 6B, hippocampus: *p* = 0.068 *vs* WT; cortex: *p* = 0.602 *vs* WT). Because tau hyperphosphorylation is the pathological feature of AD, increased hyperphosphorylated tau protein have been reported in the brains of APP/PS1 transgenic mice (Aso et al., 2012; Busche and Hyman, 2020), we then used the AT8 (PHF-tau, Ser202/Thr205) antibody to detect the level of the phosphorylated tau in this model. We found that the AT8 level was not changed in the hippocampus but increased in the temporal lobe cortex of APP/PS1 mice at the age of 6 months (Figure 6C, hippocampus: *p* = 0.05 *vs* WT; cortex: *p* = 0.002 *vs* WT). Importantly, Lv-pre-*miR-195* treatment for 2M significantly inhibited the elevated APP level in the hippocampus (Figure 6A, hippocampus: *p* = 0.022 *vs* Lv-NC) but without affecting APP in the temporal lobe cortex (Figure 6A, hippocampus: *p* = 0.393 *vs* Lv-NC) and AT8 level (Figure 6C, hippocampus: *p* = 0.830 *vs* Lv-NC; cortex: *p* = 0.665 *vs* Lv-NC) as well as BACE1 expression in both hippocampus and temporal lobe cortex (Figure 6B, hippocampus: *p* = 0.467 *vs* Lv-NC; cortex: *p* = 0.414 *vs* Lv-NC). Interestingly, compared with WT mice, qRT-PCR results showed that the *miR-195* level was not changed in the brains of APP/PS1 mice at 6M (Figure 6D, hippocampus: *p* = 0.693 *vs* WT; cortex: *p* = 0.910 *vs* WT), but were significantly increased in mice of the same age who were injected with Lv-pre-*miR-195* with 2 months (Figure 6D, hippocampus: *p* = 0.0006 *vs* Lv-NC; cortex: *p* = 0.004 *vs* Lv-NC).

**FIGURE 7:**
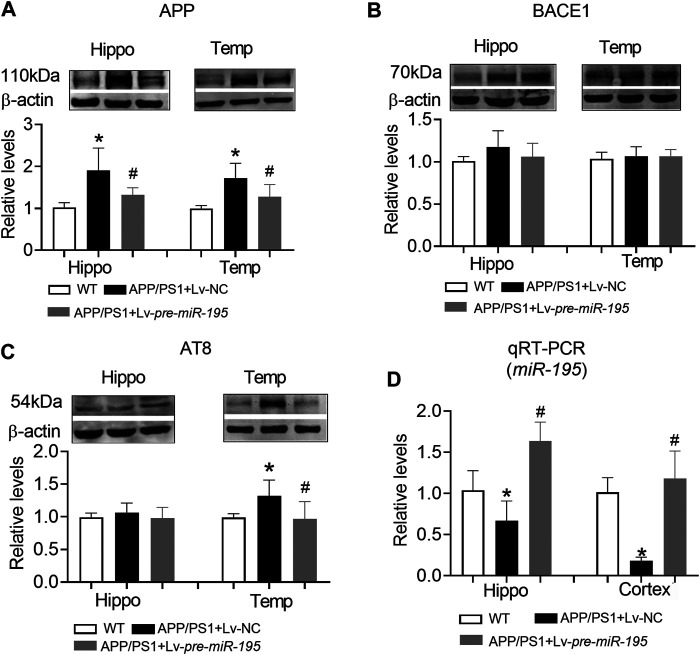
The effect of *miR-195* on the expression of APP, BACE-1, AT8 protein in the hippocampi of APP/PS1 mice after Lv-pre*-miR-195* injection for 3M **(A)** *MiR-195* down-regulated the expression of APP protein in the hippocampus and temporal lobe of APP/PS1 mice. *p < 0.05 vs. WT. #*p* < 0.05 vs. Lv-NC **(B)** There was no significant change in BACE-1 protein in hippocampus and temporal lobe of 7M-old APP/PS1 mice **(C)** *MiR-195* reverses the high expression of AT8 in the temporal lobe of 7M-old APP/PS1 mice, but not in the hippocampus. *p < 0.05 vs. WT **(D)** *MiR-195* levels in the hippocampus and cortex of APP/PS1 mice after brain injection of Lv-pre*-miR-195* for 3M. *p < 0.05 vs. WT. #*p* < 0.05 vs. Lv-NC. *n* = 6.

Similar to APP/PS1 mice at the age of 6M, APP level in both hippocampus and temporal lobe cortex were significantly increased in mice aged 7M (Figure 7A, hippocampus: *p* = 0.003 *vs* WT; cortex: *p* = 0.003 *vs* WT), but the expression of BACE1 was still not changed (Figure 7B, hippocampus: *p* = 0.109 *vs* WT; cortex: *p* = 0.608 *vs* WT). Interestingly, AT8 expression did not change in the hippocampus (Figure 7C, *p* = 0.276 *vs* WT), but increased significantly in the temporal lobe cortex (Figure 7C, *p* = 0.009 *vs* WT). Lv-pre-*miR-195* treatment effectively reversed the increase of APP level in both hippocampus and temporal lobe cortex (Figure 7A, hippocampus: *p* = 0.03 *vs* Lv-NC; cortex: *p* = 0.0001 *vs* Lv-NC) as well as the expression of AT8 in temporal lobe cortex (Figure 7C, *p* = 0.0003 *vs* Lv-NC). Importantly, we found that *miR-195* levels in hippocampus and temporal lobe cortex of 7M-old APP/PS1 mice were significantly decreased (Figure 7D, hippocampus: *p* = 0.023 *vs* WT; cortex: *p* < 0.0001 *vs* WT), while Lv-pre-*miR-195* treatment effectively increased the expression level of *miR-195* in the brain (Figure 7D).

**FIGURE 8:**
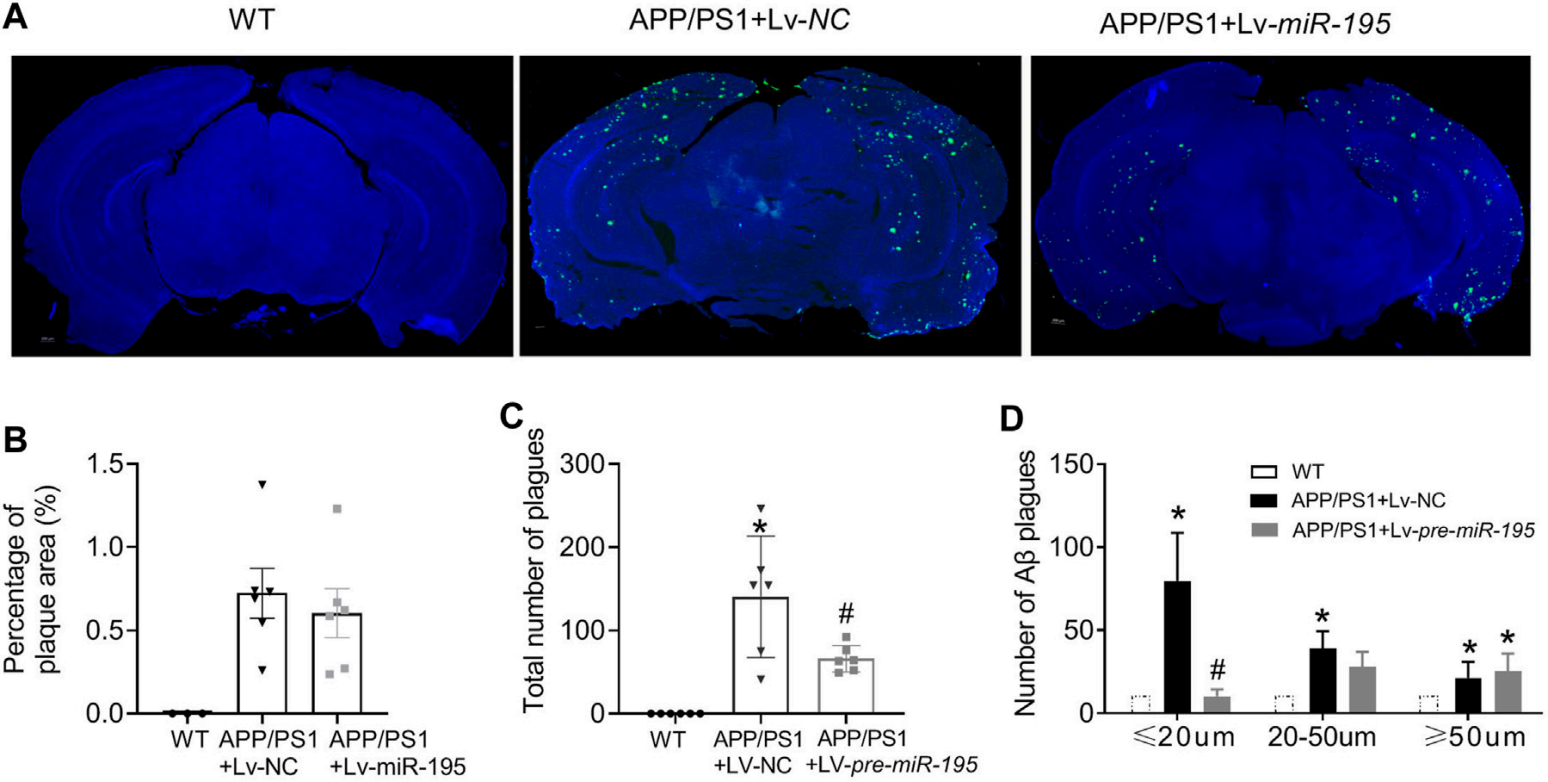
*MiR-195* reduces Aβ plaques in the brain of APP/PS1 mice **(A)** Representative confocal images showing the deposition of Aβ plaques in the brain of 7M-old WT mice, APP/PS1 mice with LV-NC injection and APP/PS1 mice with Lv-pre-*miR-19*5 injection **(B)** The percentage of Aβ plaques area in the brain of APP/PS1 mice *p < 0.05 vs. WT **(C)** The total number of Aβ plaques in the brain of APP/PS1 mice with brain injection of Lv-pre*-miR-195* for 3M. *p < 0.05 vs. WT. #*p* < 0.05 vs. Lv-NC **(D)** *MiR-195* has different effects on Aβ plaques based on different sizes (below 20 μm, 20–50 μm, above 50 μm). *p < 0.05 vs. WT. #*p* < 0.05 vs. Lv-NC.

Next, we would like to know if Lv-pre-*miR-195* treatment could reduce Aβ aggregates in the brain of 7M-old APP/PS1 mice. By performing immunofluorescence staining, we found that the total area of Aβ plaques in the brain was similar between APP/PS1 mice and APP/PS1 group treated with Lv-pre-*miR-195* (Figures 8A,B, *p* = 0.5748 *vs* Lv-NC). However, compared with that in APP/PS1 mice injected with Lv-NC, the total number of Aβ plaques in the brain of APP/PS1 mice treated with Lv-pre-*miR-195* was significantly reduced (Figure 8C, *p* = 0.035 *vs* Lv-NC). By counting the number of different size of Aβ plaques, we found that Lv-pre-*miR-195* treatment mainly affected Aβ plaques smaller than 20 μm, but did not affect Aβ plaques larger than 50 μm (Figure 8D, ≤20 μm: *p* = 0.0002 *vs* Lv-NC; 20∼50 μm: *p* < 0.0001 *vs* Lv-NC; >50 μm: *p* = 0.3286 *vs* Lv-NC). The result suggests that Lv-pre-*miR-195* treatment probably mainly inhibited the formation of new plaques but had no effect on those plaques that had already formed.

## Discussion

Although many new drugs have been developed to treat AD, many clinical trials of monotherapy treatments have failed to affect disease progression or symptoms compared with placebo (Godyn et al., 2016; Cummings et al., 2019). It has been reported that up-regulating *miR-195* could improve cognitive decline of CBH rats and ApoE4^+/+^mice by targeting multiple genes (Ai et al., 2013; Cao et al., 2020). However, if *miR-195* could effectively rescue the impaired cognition APP/PS1 transgenic mouse, an acknowledged animal model used for Aβ-related drug screening, is not yet reported. In this study, we report for the first time that lentivirus-carried *miR-195* save memory deficits in APP/PS1 mice, possibly by blocking the formation of Aβ plagues rather than clearing those that have already formed Aβ plagues. The result provides new evidence that *miR-195* would be a good candidate for future Alzheimer’s therapy.

Due to the failure of single drug therapy in clinical trial, the search for effective combination therapy has become a research hotspot (Meldolesi, 2019; Tam et al., 2019). MiRNAs are multi-target RNAs that are considered to be promising biomarkers and therapeutics for a variety of diseases (Lu and Thum, 2019; Brennan and Henshall, 2020). A series of previous studies have shown that *miR-195* is involved in dementia-related pathological processes by targeting multiple genes (Ai et al., 2013; Sun et al., 2015; Liu et al., 2016; Chen et al., 2017; Cao et al., 2020; Mao et al., 2020) and up-regulation of *miR-195* in the hippocampus and cortex could improve the cognitive function of CBH rats (Ai et al., 2013) and ApoE4^+/+^mice (Cao et al., 2020). Furthermore, clinical studies have reported that *miR-195* level is decreased in the brain of patients with acute ischemic stroke (Yang et al., 2018) and in the serum of patients with vascular dementia (VaD) (Ai et al., 2013). In the present study, we reported for the first time that gain-of-function of *miR-195* in the hippocampus and cortex effectively rescue the cognitive decline in APP/PS1 mice. All these studies indicated that *miR-195* may be a prospective candidate molecule to prevent or treat cognitive decline.

In the present study, to observe whether/how the *miR-195* improves the cognitive decline in APP/PS1 mice, we first explored the optimal timing of intervention by evaluating the deposition of Aβ plague in the hippocampus and cortex. We found that markedly Aβ plagues deposition in both hippocampus and cortex of APP/PS1 mice at the age of 4M. Therefore, we delivered *miR-195* into the bilateral hippocampus of 4M-old APP/PS1 mice by lentivirus vector, which can achieve long-term stable expression. Similar to the previous studies, we found that the cognitive phenotype of APP/PS1 mice decreased at 6M rather than 5M (Aso et al., 2012; Shen et al., 2017) and there was no change after 1 month treatment. Subsequently, we observed that Lv-pre-*miR-195* treatment with 2M improved the decline in learning ability of 6M-old APP/PS1 mice, but did not affect their impaired memory function. However, treatments with Lv-pre*-miR-195* for 3 months successfully revised the impaired cognitive decline indicated by reduced latency time in the cued learning trial and increased the crossing times in the probe trial. The data suggested that local hippocampal injection of lentivirus-mediated *miR-195* can effectively prevent cognitive decline in APP/PS1 mice at the age of 6 and 7-month old; however, medication time was at least 2 months.

The genetic basis of APP/PS1 mice is that it is a double transgenic mouse expressing a chimeric mouse/human amyloid precursor protein (APP, Mo/HuAPP695swe) and a mutant human presenilin 1 (PS1-dE9). They are directly transfected into CNS neurons and are associated with the early onset of AD. Thus, the primary pathology of this animal model was the accumulation of Aβ plagues caused by up-regulation of the APP and PS1 genes. Because previous study has shown that brain injection of the lentiviral vector-mediated antisense oligoribonucleotides *miR-195* (Lv-pre*-*AMO*-195*) can lead to impaired cognitive function and increased hippocampal Aβ deposition in normal rats, and after simultaneous injection of LV-pre*-*AMO*-195* and LV-pre*-miR-195* in the hippocampus of normal rats, LV-pre*-miR-195* reversed cognitive impairment and increased Aβ deposition in LV-pre*-*AMO*-195* rats (Ai et al., 2013). Therefore, we selected APP/PS1 mouse as the animal model to verify the role of *miR-195* by detecting the expression of the APP and BACE1 proteins, Aβ plagues and phosphorylation of Tau. We found that, after 2 months treatment of Lv-*pre-miR-195*, although the increase of APP in the hippocampus was significantly inhibited, the increase of AT8 level in the cortex was not prevented. However, Lv-*pre-miR-195* successfully inhibited the increase of APP and AT8 levels in the hippocampus and cortex after 3 months of treatment. More notably, the expression of BACE1 in the brain of APP/PS1 mice at 6 and 7M of age did not change, nor did Lv-pre-*miR-195* affect its expression. Our previous study found that miRNA showed competitive binding with different target genes (Ai et al., 2013). Since the expression level of APP in the hippocampus of APP/PS1 mice was higher than normal, while the expression of BACE1 did not change, we speculated that *miR-195* would preferentially bind APP to play its role, and there was no excess *miR-195* that could bind BACE1 gene to regulate the expression of BACE1 protein. Taken together, these results indicated that gain-of-function of *miR-195* rescued cognitive decline of APP/PS1 mice that related to preventing the increase of APP expression but not affecting BACE1 level.

We know that Aβ plague aggregation depends on the overexpression level of APP, and down-regulation of APP protein expression can prevent Aβ deposition. Therefore, treatment with Lv-*pre*-*miR-195* is predicted to affect Aβ deposition in the brain of APP/PS1 mice. By quantitative analysis, compared with APP/PS1 mice treated with Lv-NC, we found that the total area of Aβ plaques in the brain of APP/PS1 mice treated with Lv-pre-*miR-195* did not change; however, the total number of Aβ was significantly reduced. The further analysis revealed that the phenomenon was contributed by the significant reduction of Aβ plagues with Aβ diameter of less than 20 μm in Lv-pre-*miR-195*-treated APP/PS1 mice, which was not plentiful enough to affect the percentage of plagues area. These results indicated that the gain-of-function of *miR-195* could block Aβ plagues production, but could not eliminate Aβ plagues formed in APP/PS1 mice.

The consensus is that the Aβ plagues deposition occurs 10–20 years before the onset of dementia. Preventing the formation and aggregation of Aβ plagues is the best strategy to prevent cognitive decline. In this study, although we reported for the first time that the gain-of-function of *miR-195* can effectively improve the cognitive performance of APP/PS1 mice by preventing Aβ plagues generation, whether the effect of *miR-195* on cognitive function in APP/PS1 mice is also related to activated microglia and reactive astrocytes or other markers of neuroinflammation remains to be further investigated. In addition, in the present study, the way of the drug application was the stereotaxic injection of the lentiviral vectors to hippocampus. However, the administration strategy of local brain injection is nearly impossible in clinic; and brain-targeted therapy with intravenous or oral lentiviral-carried nucleic acid molecules (such as miR-195) remains to be a very difficult technical hurdle. Therefore, it would be very important to develop and applicate some new nucleic acid drug carriers to provide guarantee for the clinical application of nucleic acid drugs.

## Data Availability

The original contributions presented in the study are included in the article/Supplementary Material, further inquiries can be directed to the corresponding authors.
