# Supplementary material for: Lentivirus-Carried microRNA-195 Rescues Memory Deficits of Alzheimer’s Disease Transgenic Mouse by Attenuating the Generation of Amyloid Plaques
Source: Front Pharmacol. 2021 Apr 26;12:633805. doi: 10.3389/fphar.2021.633805 (PMC8109030; doi:10.3389/fphar.2021.633805)
Supplement: Supplementary file 1 [file DataSheet1.pdf]

Figure 6A APP Hippo

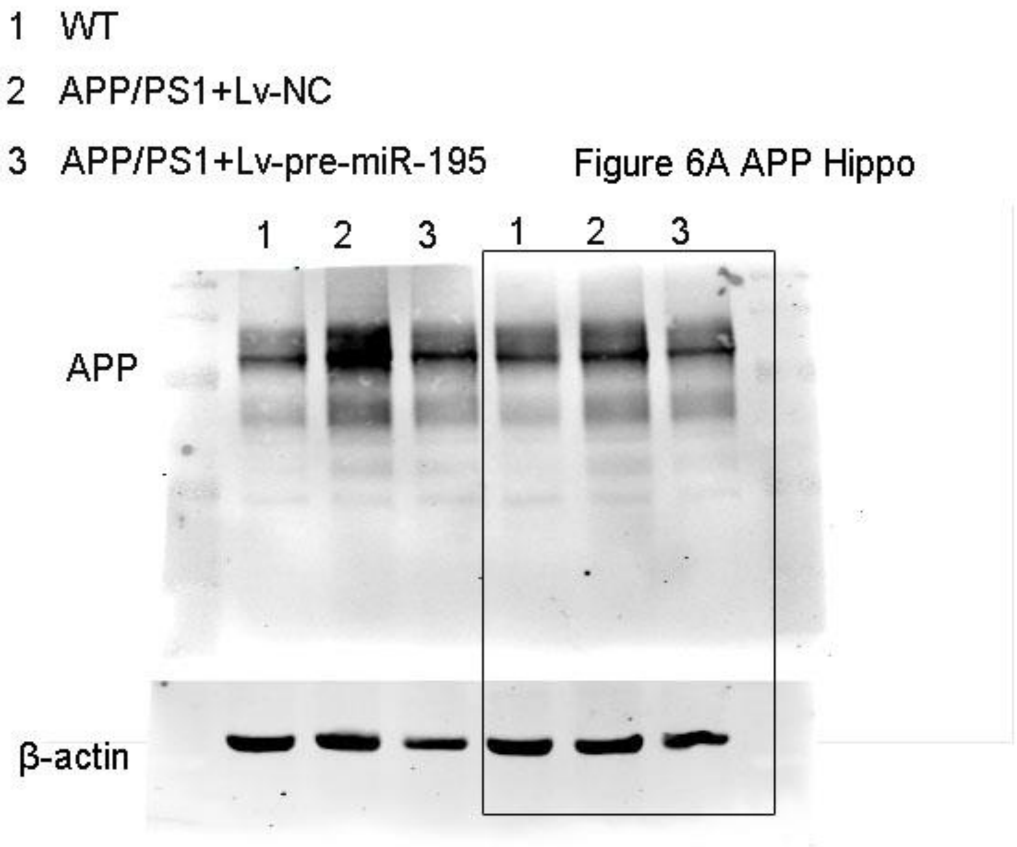

Figure 6A APP Temp

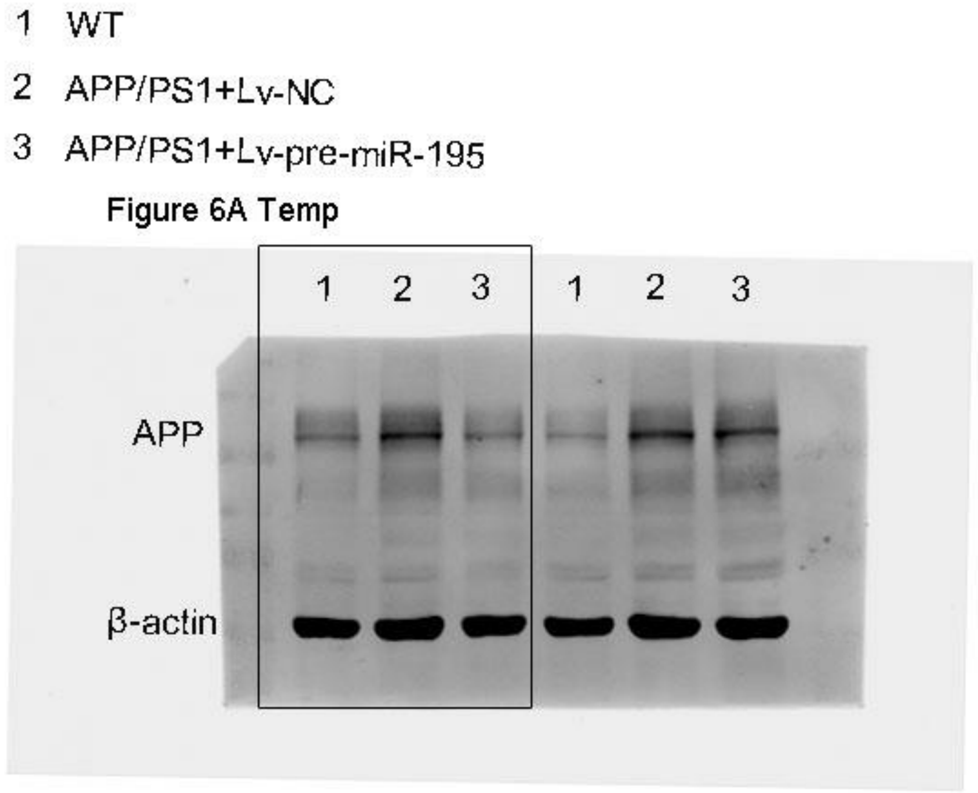

Figure 6B BACE1 Hippo

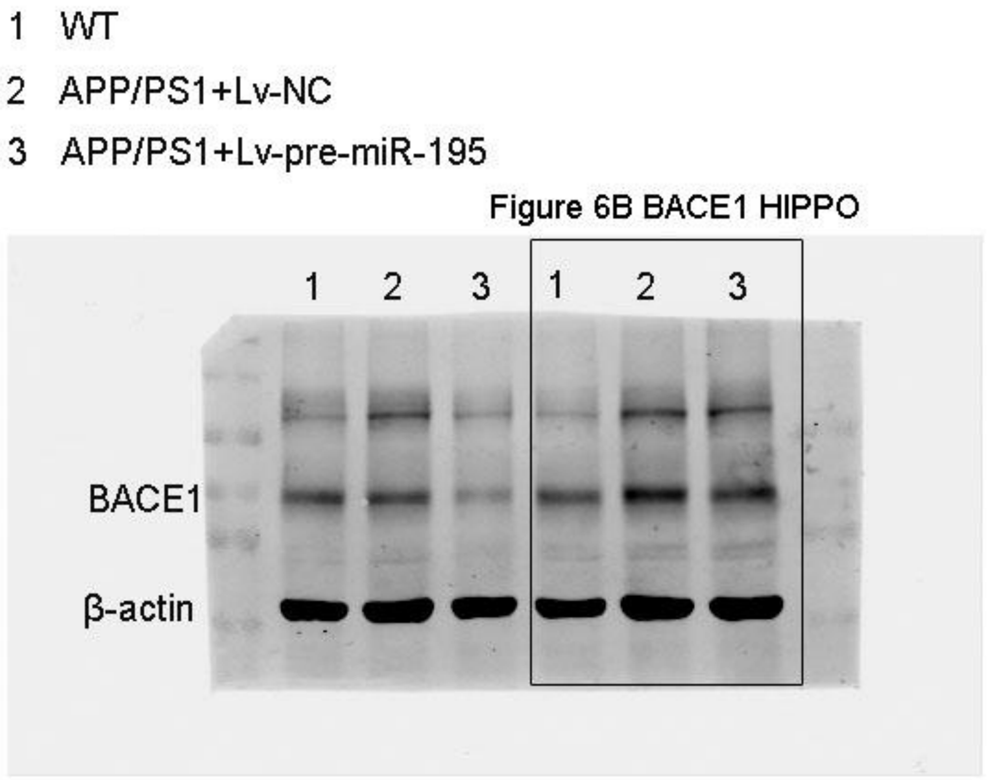

Figure 6B BACE1 Temp

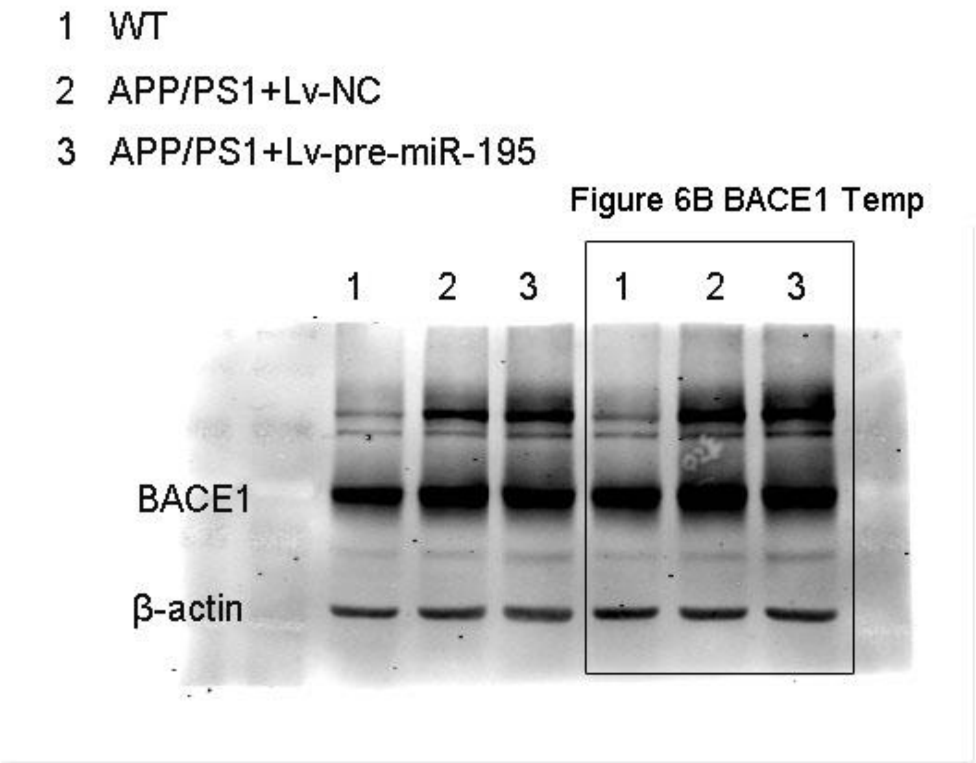

Figure 6C AT8 Hippo

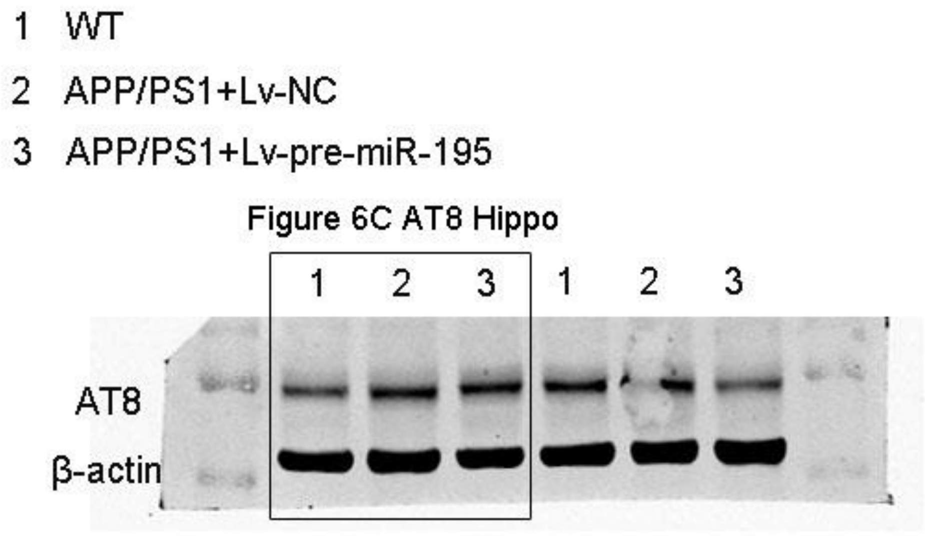

Figure 6C 7C   AT8 Temp

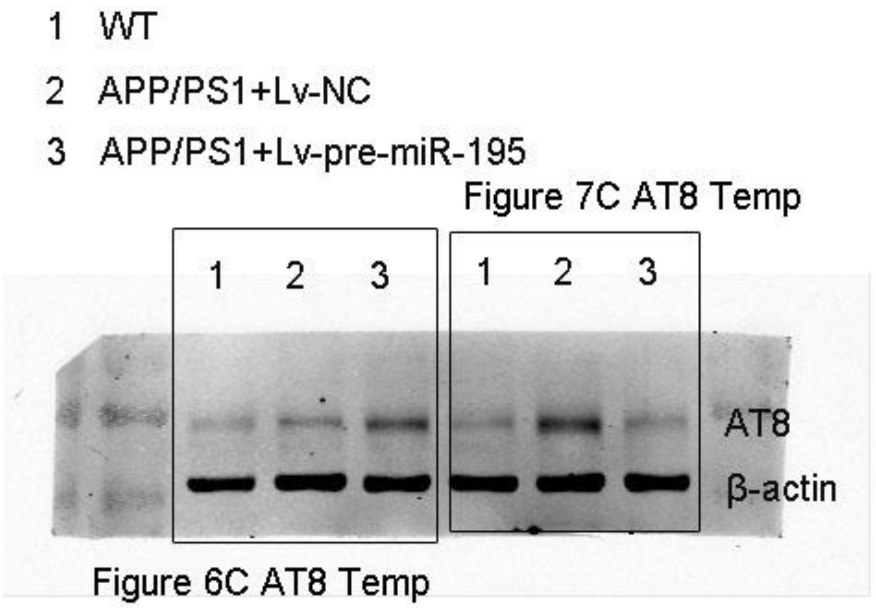

Figure 7A APP Hippo

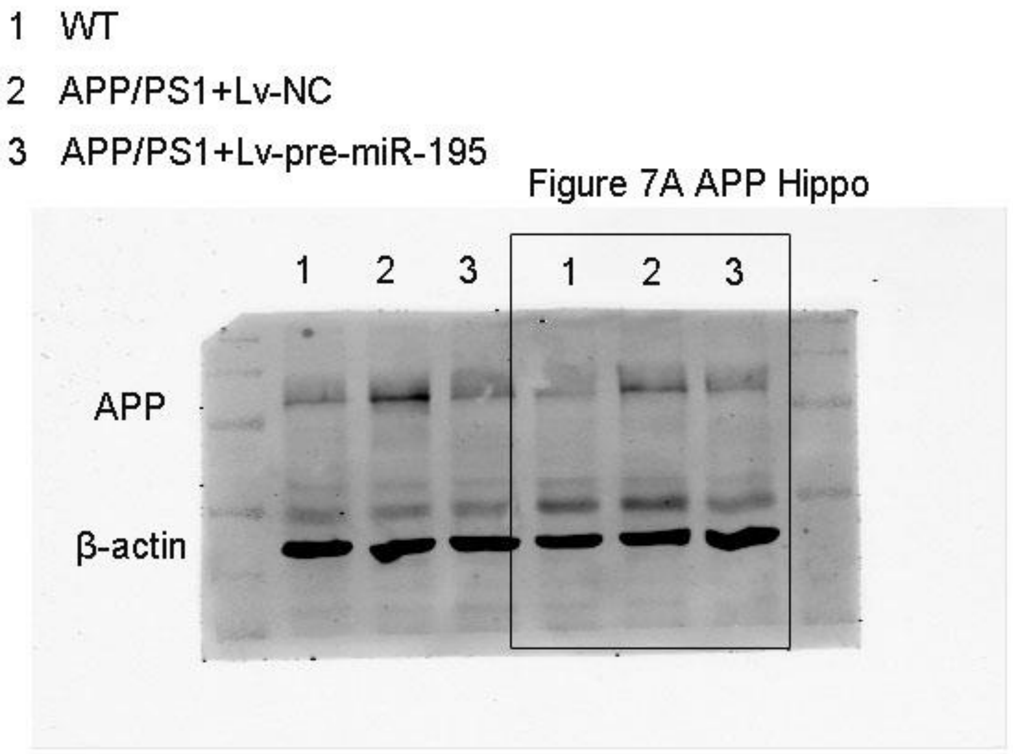

Figure 7A APP Temp

1 WT

2 APP/PS1+Lv-NC

3 APP/PS1+Lv-pre-miR-195

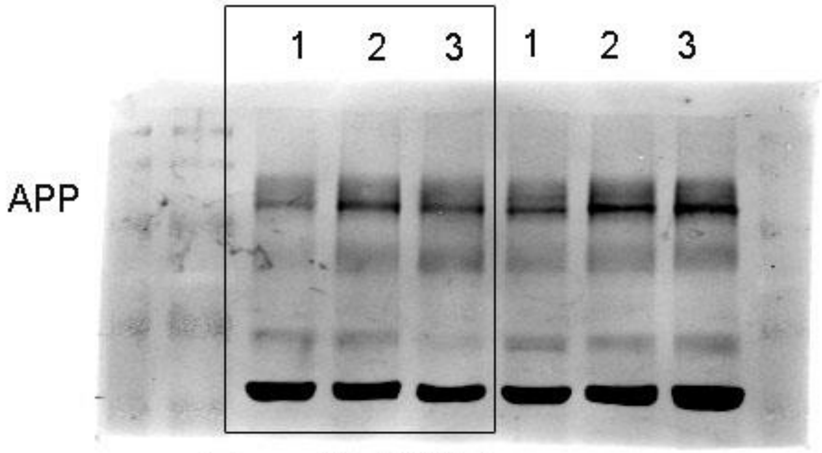

Figure 7A APP Temp

Figure 7B   BACE1 Hippo

1 WT

2 APP/PS1+Lv-NC

3 APP/PS1+Lv-pre-miR-195

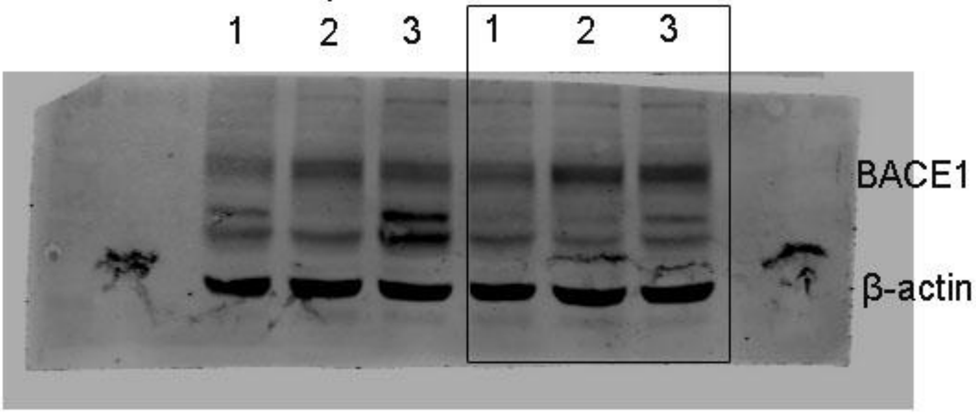

Figure 7B BACE1 Hippo

Figure 7B   BACE1 Temp

1 WT

2 APP/PS1+Lv-NC

3 APP/PS1+Lv-pre-miR-195

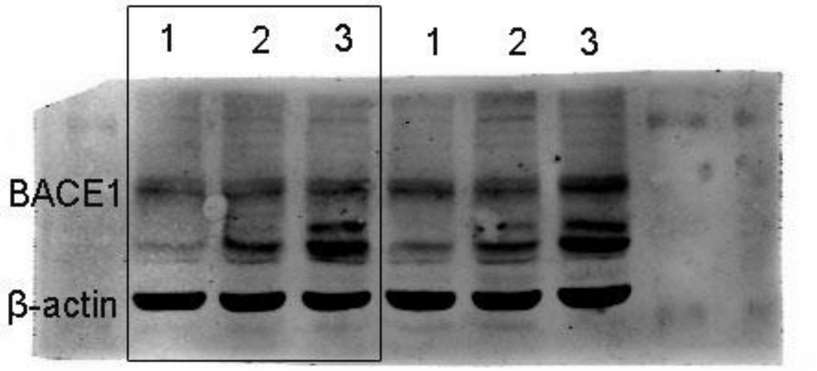

Figure 7B BACE1 Temp

Figure 7C AT8 Hippo

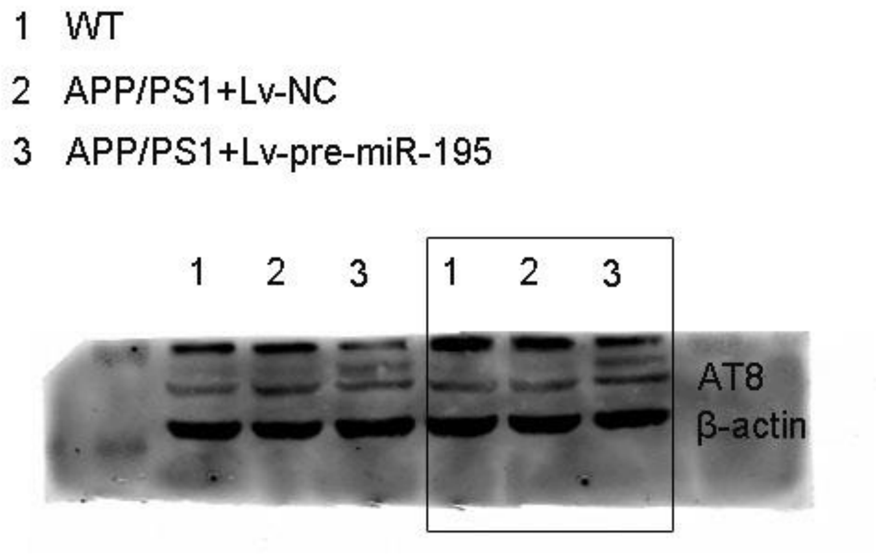

Figure 7C AT8 Hippo
